# Identification of endophytic *Bacillus mojavensis* with highly specialized broad spectrum antibacterial activity

**DOI:** 10.1007/s13205-016-0508-5

**Published:** 2016-09-01

**Authors:** B. Jasim, S. Sreelakshmi, Jyothis Mathew, E. K. Radhakrishnan

**Affiliations:** 1 School of Biosciences, Mahatma Gandhi University, PD Hills (PO), Kottayam, 686 560 Kerala India; 2 Inter University Instrumentation Centre, Mahatma Gandhi University, PD Hills (PO), Kottayam, 686 560 Kerala India

**Keywords:** Endophytic *Bacillus mojavensis*, *Bacopa monnieri*, Lipopeptide antibiotics, LC–MS/MS, PCR based genome mining

## Abstract

Biosynthetic adaptation of endophytic bacteria to chemically support host plant is very remarkable. Hence these organisms from medicinal plants are considered as highly valuable sources for natural products with diverse bioactivity. Their metabolite diversity and biosynthetic versatility have been increasingly explored for drug discovery. In this study, an endophytic *Bacillus mojavensis* with broad spectrum antibacterial properties has been analyzed for the chemical basis of its activity. By LC–MS/MS the organism was identified to have the biosynthetic ability to produce lipopeptides surfactin and fengycin. The impressive antibacterial activity of *B. mojavensis* as reported in the study indicates its broad antimicrobial applications.

## Introduction

Endophytic bacteria and host plant mutualistically support each other to manage various environmental stresses. Biodiversity richness and biosynthetic versatility of endophytes make them a treasure trove of unexplored natural products (Jasim et al. 2013a). Microbial production of diverse chemical scaffolds with broad phyto-interactive or protective properties may expect to provide preference for their endophytic life. Identification of such promising isolates can have immense applications in various fields ranging from clinical, industry to agriculture.

One of the most important bacterial groups with common endophytic association with plants and heavy deposition of biosynthetic pathway include those of the *Bacillus* sp. Various species of *Bacillus* have been reported to have remarkable distribution of lipopeptide compounds synthesized by non-ribosomal peptide synthase (NRPS) (Moyne et al. 2004). These compounds have a common amphipathic structure with a hydrophilic peptide and a hydrophobic fatty acid region. The lipopeptide compounds synthesized by *Bacillus* spp. are considered to have the potential to manage infections caused by multidrug resistant and biofilm forming pathogens (Fracchia et al. 2015; Meena and Kanwar 2015). Rapid development of microbial resistance against lipopeptides is prevented by direct membrane binding based mechanism of action of these compounds (Straus and Hancock 2006). Because of these, exploration of novel sources for bacterial strains with multipotent lipopeptide synthesizing property is very important. This property has also been demonstrated in relatively new members of genus *Bacillus* like *B. mojavensis.* They differ from the related *Bacillus subtilis* by fatty acid composition, DNA sequence and resistance to genetic transformation (Bacon and Hinton 2011). They have been reported to form surfactin, iturin and fengycin group of lipopeptide as basis of its antimicrobial effects (Mounia et al. 2014). Among these compounds, broad spectrum antibacterial activity have been described for surfactin and antifungal activity for fengycin.

*Bacillus mojavensis* associated endophytically with medicinal plants can expect to have promising lipopeptide synthesizing property. Hence, *B. mojavensis* isolated from the plant *Bacopa monnieri* (L.) was selected as organism for the current study. The plant is used in traditional medicinal formulations and is having different types of bacopasides, stigmasterol and other saponins as its active constituents. The endophytic *B. mojavensis* isolated from the plant was observed to have broad antibacterial activity towards gram positive and gram negative organisms but was without any antifungal activity. Interestingly, the organism was positive for surfactin biosynthetic gene in PCR analysis. Further LC–MS/MS based analysis confirmed it to have the ability to synthesize surfactin (M+H^+^—1008.6602, 1022.6755) and fengycin (M+H^+^—1491.8195, 1477.8055) derivatives. This makes the study significantly interesting to explore the application of *B. mojavensis* in various fields.

## Materials and methods

### Isolation and identification of endophytic bacteria with antibacterial activity

Endophytic bacteria isolated from stem of *B. monnieri* were checked for its antibacterial activity against *Escherichia coli*, *Salmonella enterica Typhi*, *Bacillus subtilis*, *Klebsiella pneumoniae* and *Staphylococcus aureus* using perpendicular streak method. For this, the isolates obtained were initially grown for 5 days at the centre of nutrient agar plate as single line streak. After incubation, the pathogens selected were inoculated perpendicular to the isolate and incubated at 37 °C for overnight and observed for any inhibition.

The bacterial isolate BmB 4 which showed broad antimicrobial activity was further subjected to molecular identification. Bacterial genomic DNA from the selected isolate was purified using Chromous Biotech Bacterial Genomic DNA Mini Spin Kit (RKT 17) and was further subjected to PCR amplification of 16S rDNA (Jasim et al. 2013b). The sequence data of 16S rDNA was then used for BLAST and phylogenetic analysis (Zhang et al. 2000).

### Screening for natural product biosynthetic gene clusters by PCR method

*Bacillus mojavensis* BmB 4 was screened for natural product biosynthetic gene cluster by PCR based method. For surfactin biosynthetic gene clusters the primer P17 F (5′-ATg AAg ATT TAC ggA ATT TA-3′) and P18 R (5′-TTA TAA AAg CTC TTC gTA Cg-3′) were used as per the methods described previously (Tabbene et al. 2011). The amplified PCR product was checked by agarose gel electrophoresis, sequenced and was further subjected to BLAST analysis.

### Preparation of culture extract and identification of compounds by LC–MS/MS

For this, *B. mojavensis* was cultured in 3 L nutrient broth for 10 days at 30 °C in an orbital shaking incubator. The culture supernatant was extracted twice using double the volume of ethyl acetate and dried in a rotary vacuum evaporator at 43 °C (Jasim et al. 2014). The dried powder reconstituted in methanol was used to confirm antibacterial activity against *E. coli*, *S. Typhi*, *B. subtilis*, *K. pneumoniae* and *S. aureus* by well diffusion method. Same volume of pure methanol was used as the control.

The extract was then used for LC–MS analysis followed by LC–MS/MS analysis as per previous methods using Acquity H-Class (Waters) UPLC with BEH C18 column (50 mm × 2.1 mm × 1.7 μm) and Xevo G2 (Waters) Quadruple Time of Flight (Q-TOF) Mass spectrometer (Kumar et al. 2013).

## Results and discussion

Endophytic microorganisms have been described to have diverse chemical means to support plant to manage both biotic and abiotic stress. Presence of bio-interactive natural products in endophytes and its diversity make it to have immense applications in agricultural, industrial and medical fields. Current study selected *B. monnieri* as the source for isolation of endophytic bacteria due to its diverse medicinal applications. The plant has remarkable ability to produce biomass with in short time in addition to its medicinal properties. This also makes it reasonable to expect the endophytic microbiome of *B. monnieri* to have candidates with impressive biosynthetic potential. So the endophytic *B. mojavensis* obtained from *B. monnieri* was studied in detail to unravel the chemical basis of its antibacterial activity.

### Isolation and identification of endophytic bacteria

**Fig. 1:**
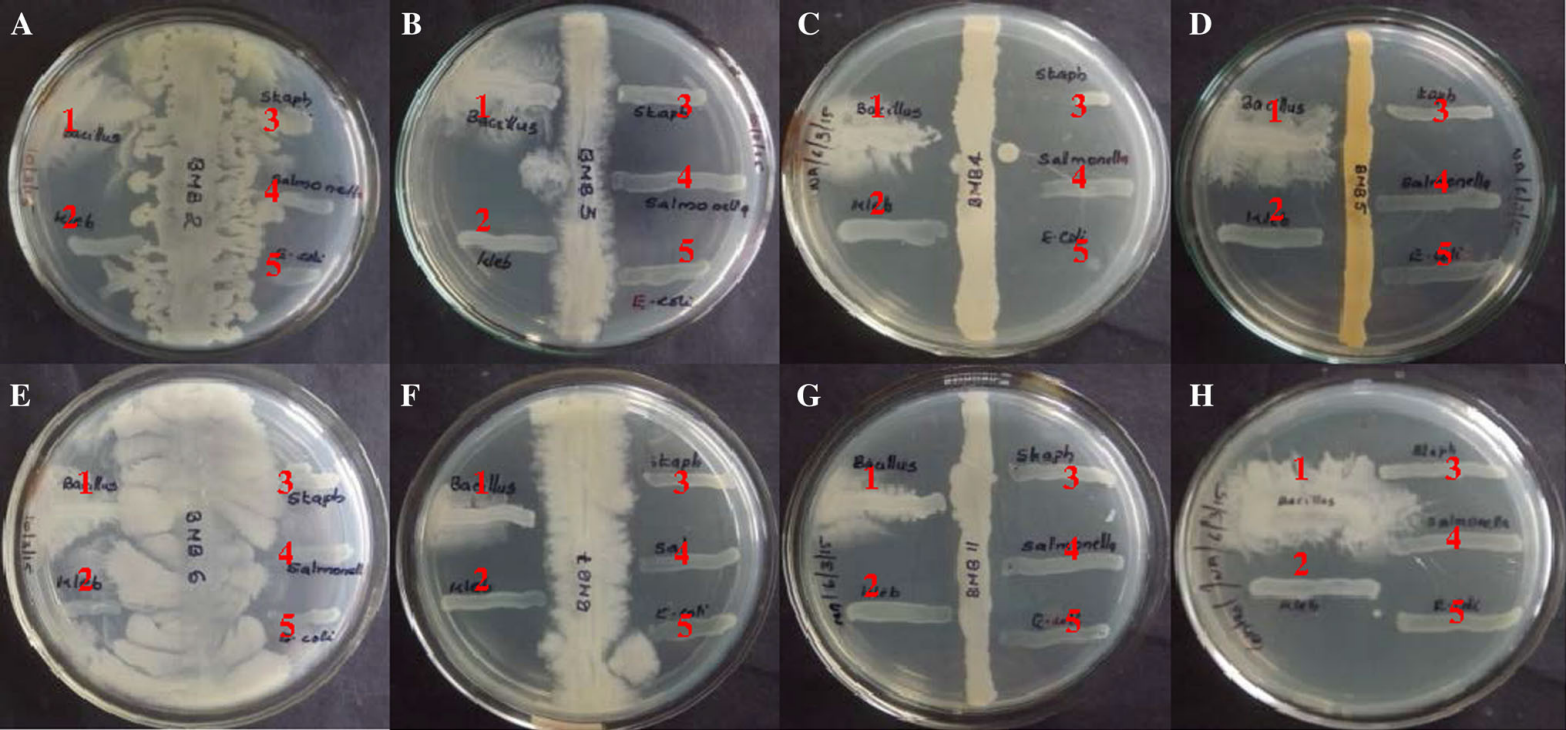
Screening of the antibacterial activity of the endophytic bacteria isolated from *Bacopa monnieri* using cross streak method. **a** Isolate BmB 2, **b** isolate BmB 3, **c** isolate BmB 4, **d** isolate BmB 5, **e** isolate BmB 6, **f** isolate BmB 7, **g** isolate BmB 11, **h** control; 1, *Bacillus subtilis*; 2, *Klebsiella pneumoniae*; 3, *Staphylococcus aureus*; 4, *Salmonella enterica Typhi*; 5, *Escherichia coli*

After purifying the endophytic bacteria from the plant tissue, the isolates were screened for antimicrobial properties. Interestingly the bacterial isolate designated as BmB 4 was found to have inhibitory effect on all the tested bacterial pathogens. This included both gram positive and gram negative organisms like *E. coli*, *S. Typhi*, *B. subtilis*, *K. pneumoniae* and *S. aureus* (Fig. 1). Hence, BmB 4 was selected for molecular identification.

**Fig. 2:**
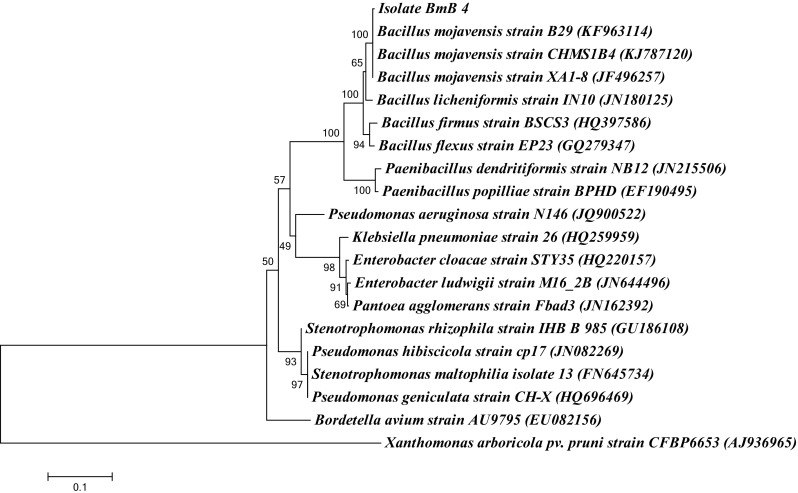
The phylogenetic analysis of 16S rDNA sequence of the endophytic bacterial isolate BmB 4 from *Bacopa monnieri* along with sequences from NCBI using MEGA 6 with neighbor joining method using 1000 bootstrap replicates

The PCR amplification product of 16S rDNA of BmB 4 has resulted in a single predominant band at 1500 bp. Further sequence analysis by BLAST showed the 16S rDNA of BmB 4 to have 99 % identity to *Bacillus mojavensis.* Its distinct clustering to same was also observed in phylogenetic analysis (Fig. 2). *B. mojavensis* isolates with significant bioactivity have already been reported from different sources including soil from dessert. Studies of Bacon et al. (2006) have previously reported the endophytic nature of *B. mojavensis* in maize with significant activity against *Fusarium* sp. Other than this, only limited reports are there on the endophytic distribution of *B. mojavensis.* This suggests the significance of exploration of this species from medicinally important plants. Remarkably the *B. mojavensis* BmB 4 was identified to be highly specialized for broad spectrum antibacterial activity. It was different from previous *Bacillus* spp. reported from our previous study due to the lack of antifungal activity. Also the values observed for the zone of inhibition caused by *B. mojavensis* in the study against the tested clinical pathogens were significantly high (Table 1).

**Table 1:** Inhibition zone shown by BmB 4 extract towards pathogens incubated at 24, 48 and 72 h (zone in mm)

Pathogens selected	Hours treated		
	24 h	48 h	72 h
*S. Typhi*	31	35	30
*K. pneumoniae*	21.5	22.5	21
*B. subtilis*	24	28	26
*E. coli*	21	26	25
*S. aureus*	36	39.5	30

### Screening for natural product biosynthetic gene clusters by PCR method

**Fig. 3:**
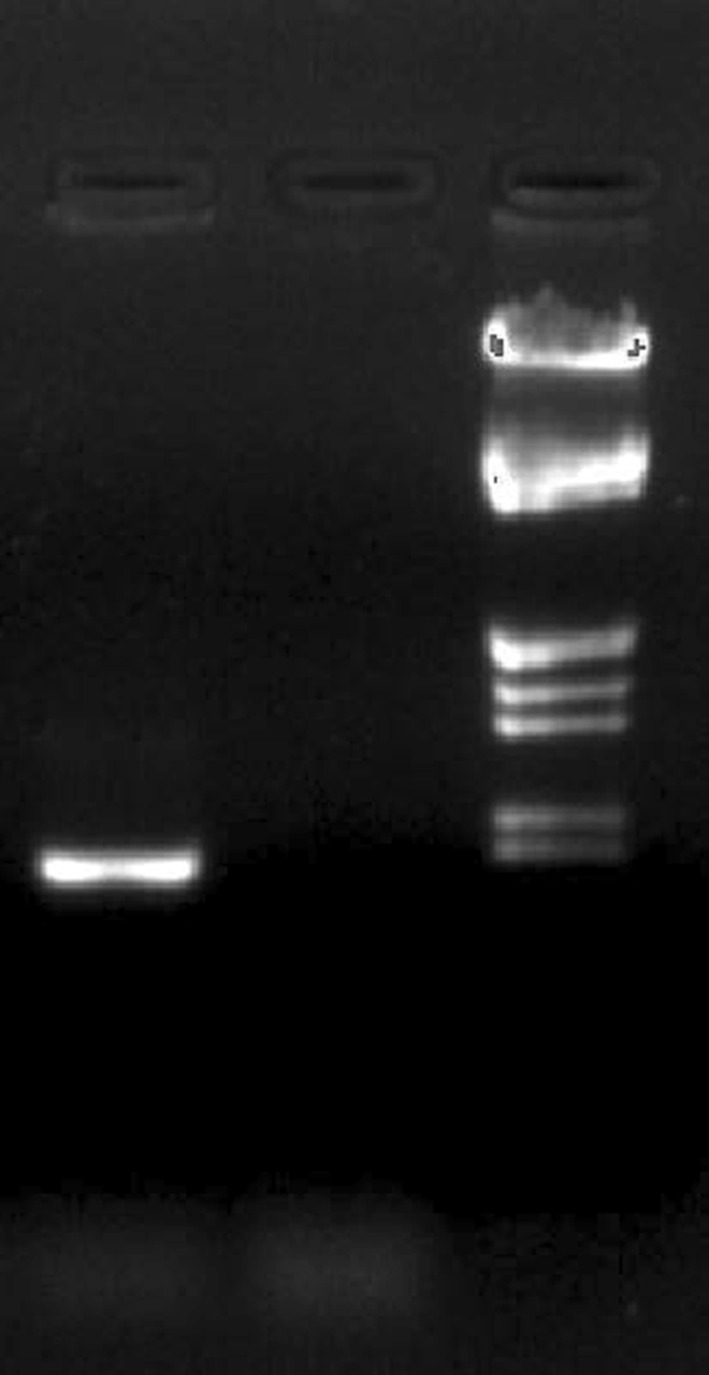
PCR screening of surfactin biosynthetic potential of endophytic bacterial isolate BmB 4 from *Bacopa monnieri*

**Fig. 4:**
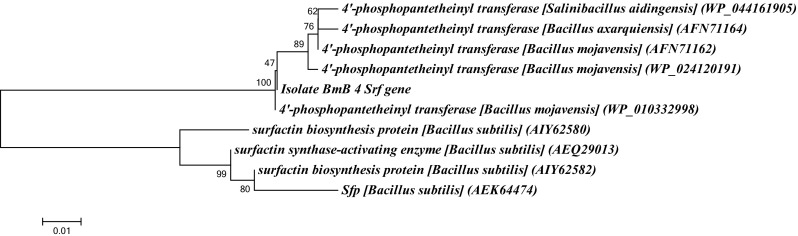
The phylogenetic analysis of surfactin (*srf*) gene sequence of the endophytic bacterial isolates BmB 4 along with sequences from NCBI using MEGA 5 with neighbor joining method using 1000 bootstrap replicates

For the rapid screening of genetic basis of antibacterial activity of BmB 4, it was subjected to PCR using primers specific to various natural product biosynthetic gene clusters. Interestingly, BmB 4 was found to be positive for surfactin (*srf*) biosynthesis gene due to the formation of a product with the expected size of 645 bp (Fig. 3). Upon BLAST analysis of sequence data, it showed 100 % identity towards 4′-phosphopantetheinyl transferase of *B. mojavensis* (WP_010332998) and the same was reflected in the phylogenetic analysis also (Fig. 4). Thus, *B. mojavensis* BmB 4 was confirmed to have the genomic basis for surfactin biosynthesis. Its presence in the medicinal plant *B. monnieri* as an endophyte adds its significance as it is very reasonable to consider its biosynthetic potential to be favorable for the plant.

### Confirmation of antibacterial activity

**Fig. 5:**
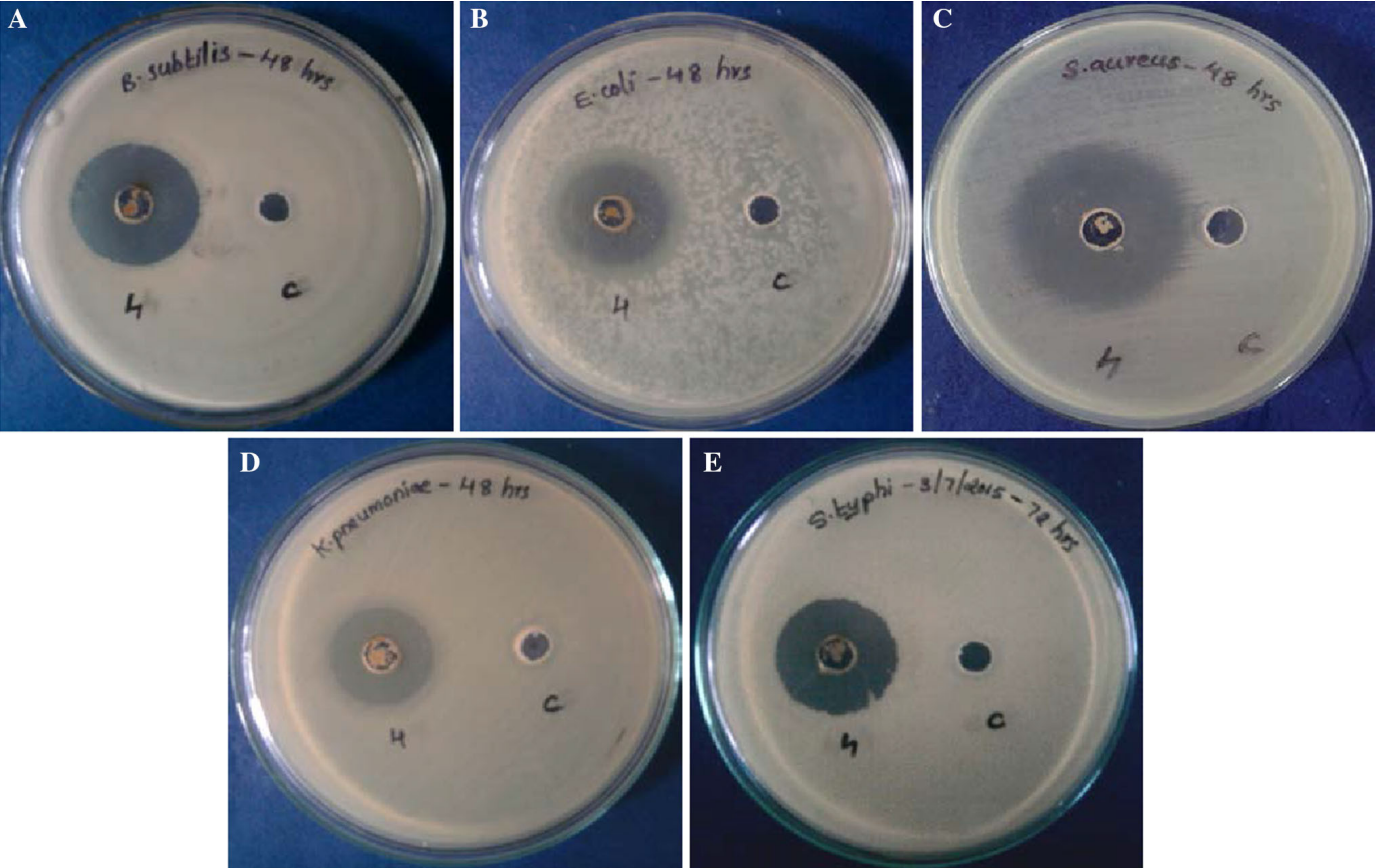
Confirmation of antibacterial property using the crude methanolic extract of the selected endophytic bacterial isolate BmB 4 from *Bacopa monnieri.* **a** *Bacillus subtilis*; **b** *Escherichia coli*; **c** *Staphylococcus aureus*; **d** *Klebsiella pneumoniae*; **e** *Salmonella enterica Typhi*

For this, the crude extract from BmB 4 was checked for activity against *E. coli*, *S. Typhi*, *B. subtilis*, *K. pneumoniae* and *S. aureus*. The extract from BmB 4 was found to have high inhibition towards most of the pathogens tested (Fig. 5). This is of particular interest as the test organisms selected included both gram positive and gram negative organisms. So *B. mojavensis* isolated in the study can be confirmed to have the chemical means to act against broad range of bacterial pathogens. Upon time-dependent analysis of activity of extract of BmB 4, the activity against test pathogens was found to be high at 48 h incubation, compared to that at 24 and 72 h (Table 1). This also make it to be an impressive candidate organism for natural product study. Based on PCR results of the study, presence of surfactin as one of the promising chemicals was also expected. To further confirm this, the extract from BmB 4 was subjected to detailed LC–MS analysis.

### LC–MS analysis of extract from *B. mojavensis* BmB 4

**Fig. 6:**
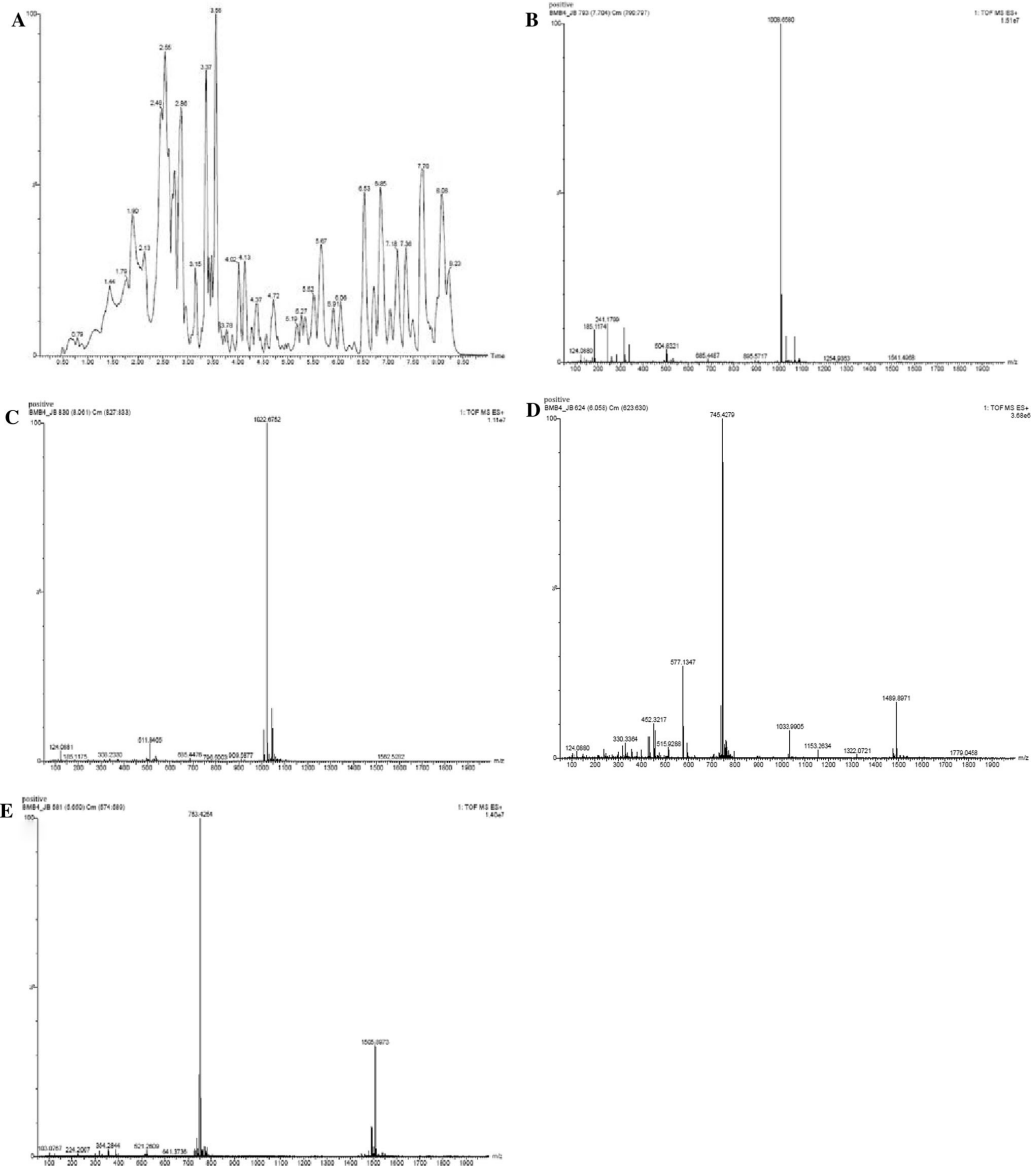
LC–MS analysis of the extract of endophytic isolate BmB 4. **a** LC chromatogram; **b** MS spectra of *m/z* 1008.6580; **c** MS spectra of *m/z* 1022.6752; **d** MS spectra of *m/z* 1489.8971; **e** MS spectra of *m/z* 1505.9019

Before the LC–MS analysis, the crude methanolic extract prepared from *B. mojavensis* was checked for its activity against the selected pathogens by well diffusion method. This confirmed the presence of antimicrobial compounds in the prepared extract. LC–MS analysis of the extract showed the presence of surfactin and fengycin derivatives with *m/z* of 1008.6580 and 1022.6752 (M+H^+^) and *m/z* 1489.8971 and 1505.9019 (M+H^+^), respectively, in the extract. Presence of same mass in positive and negative mode ionization was considered as confirmation of presence of surfactin and fengycin in the extract (Fig. 6).

**Fig. 7:**
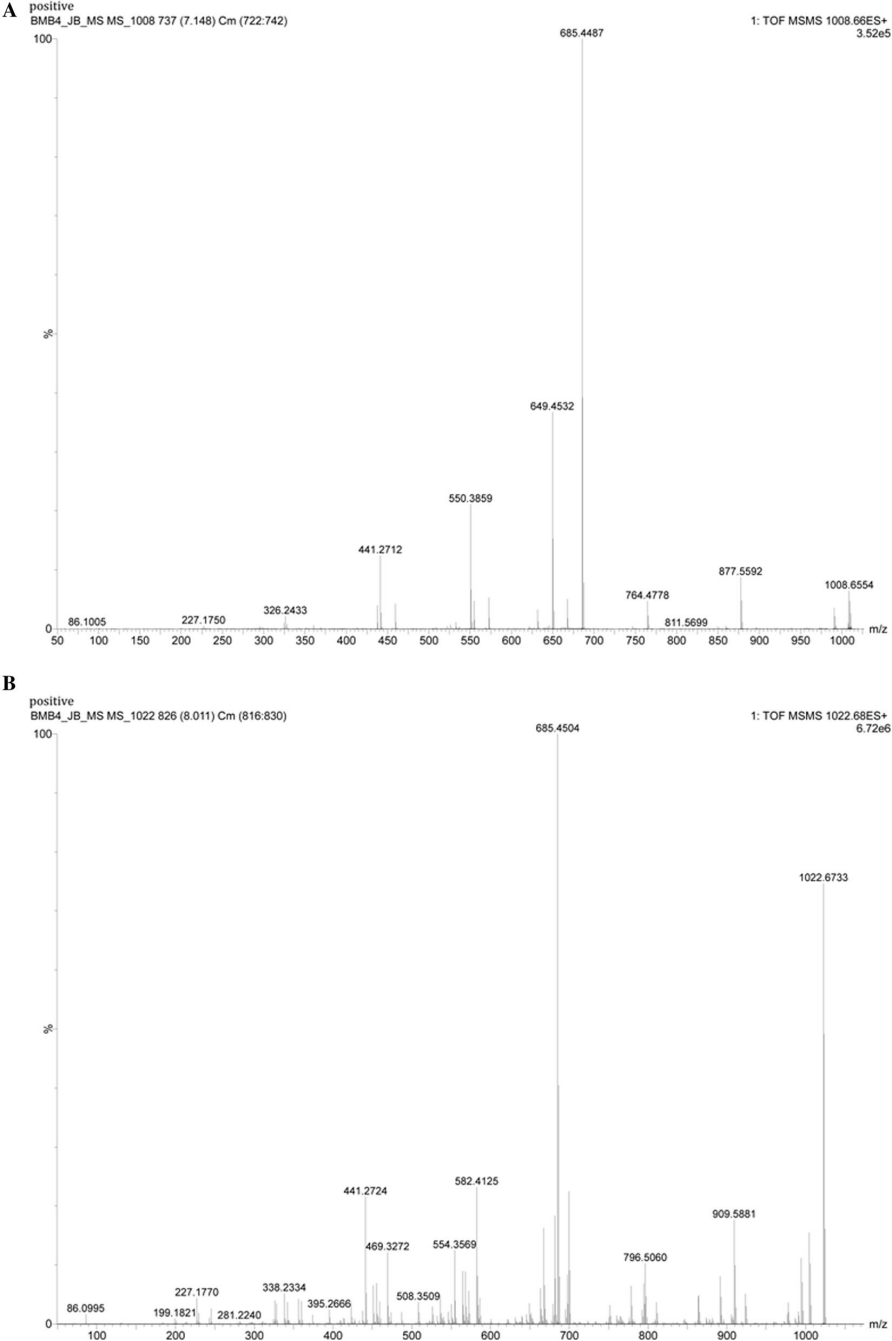
LC-MS/MS based fragmentation analysis surfactin (*m/z* 1008.66 and 1022.68) of extract of isolate BmB 4. **a** Mass spectra of *m/z* 1008.66 showing specific fragments *m/z* 990, 877, 685, 554 and 441. **b** Mass spectra of *m/z* 1022.68 showing specific fragments *m/z* 1004, 909, 685, 554 and 441

In the case of surfactin (1008.6 and 1022.6), the detailed LC–MS/MS analysis of *m/z* 1008.6 showed the presence of fragmented masses 990, 877.5592, 685.4487, 554.3859, 441.2712 and it confirmed the compound as surfactin. For *m/z* of 1022.6 the fragmentation analysis masses 1004, 909.5881, 891, 685.4504, 554.3569, 441.2724 was confirmatory to its identity as surfactin (Fig. 7; Table 2). Interestingly, the presence of key mass 685 was found to be conserved in both cases (Tang et al. 2010).

**Table 2:** Summary of the masses identified along with the fragmented masses by LC–MS/MS analysis

No.	Compounds identified	Retention time	Molecular ions (*m/z*)		Fragmented mass (*m/z*)
			[M+H^+^]	[M−H^−^]	
1.	Surfactin derivatives	7.998	1008.6580	1006.6470	990, 877.5592,685.4487, 554.3859, 441.2712
		8.061	1022.6752	1020.6615	1004, 909.5881,891, 685.4504, 554.3569, 441.2724
2.	Fengycin derivatives	6.058	1489.8971	1487.3312	1108, 994
		6.851	1505.9019	1503.8871	1108.5684, 966 675.4319

**Fig. 8:**
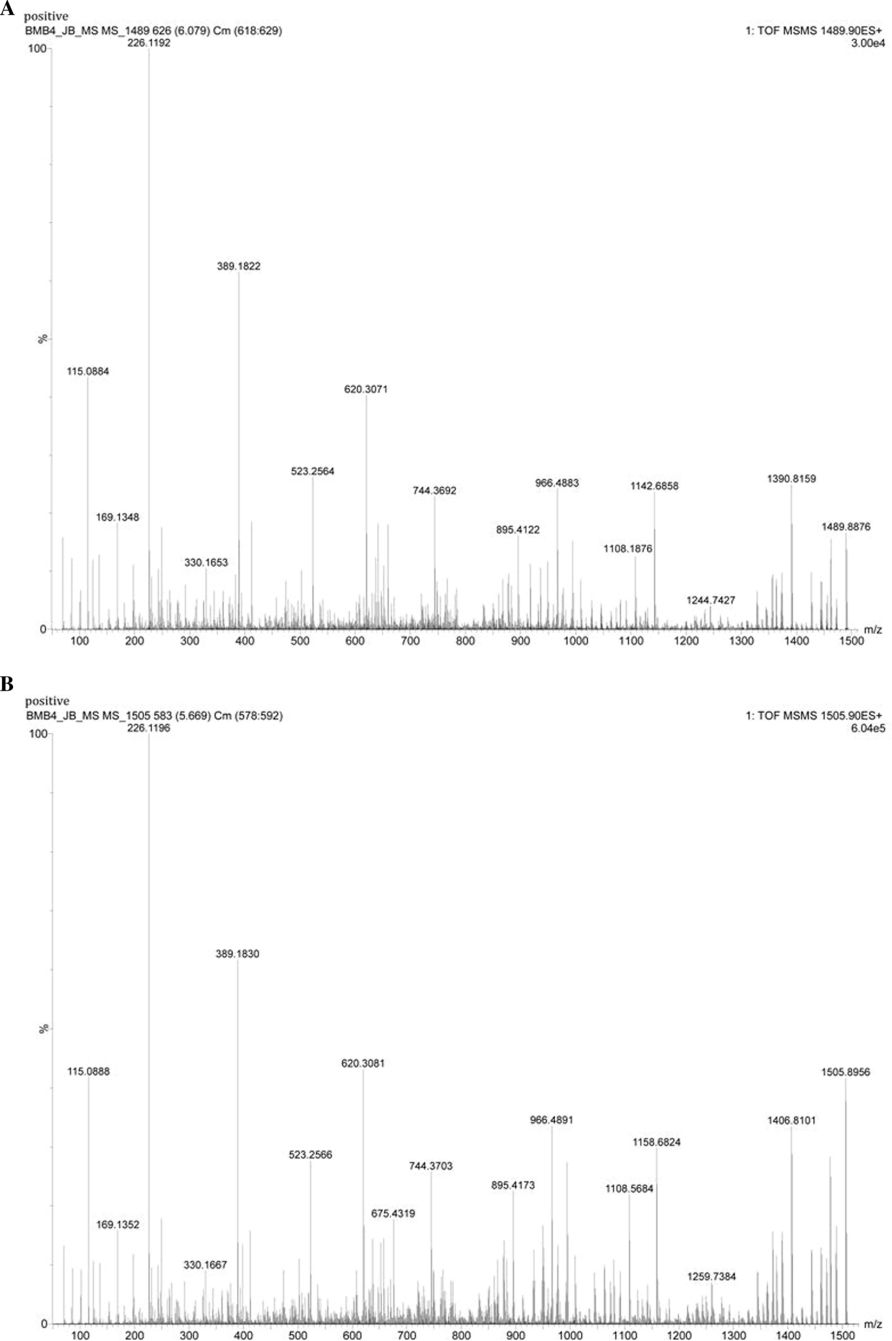
LC-MS/MS based fragmentation analysis fengycin (*m/z* 1489.90 and 1505.89) of extract of isolate BmB 4. **a** Mass spectra of *m/z* 1489.90 showing specific fragments *m/z* 1108 and 966. **b** Mass spectra of *m/z* 1505.89 showing specific fragments *m/z* 1108, 966 and 675

Fengycin was also identified due to the presence of masses 1491.8195 and 1505.9019 in the initial MS analysis. The fragmentation of 1489.8971 (M+H^+^) gave masses 1080, 966 and 675 specific to fengycin B (Fig. 8; Table 2). Likewise, fragmentation of *m/z* 1505.9019 proved the presence of masses 1108 and 994 to confirm it as fengycin. The detailed mass fragmentation analysis confirmed the chemical basis of broad antimicrobial activity of BmB 4 as due to surfactin and fengycin.

Several strains of *B. subtilis* have been reported to produce a range of antimicrobial cyclic lipopeptides including fengycins and surfactins with broad antimicrobial properties (Arrebola et al. 2010). *B. mojavensis* isolated from diverse environments have been reported to have significant activity against phytopathogens also (Özyilmaz and Benlioglu 2013). But *B. mojavensis* BmB 4 isolated in the study was not found to have any activity against phytopathogens when it was tested and it might have specialised for broad antibacterial activity as part of its endophytic adaptation. Fengycin group of compounds produced by *B. mojavensis* have been reported to have high antimicrobial activity (Mounia et al. 2014). However, its endophytic origin makes the current result highly impressive. Fengycins are synthesized nonribosomally by peptide synthetases encoded by *fenC*, *fenD*, *fenE*, *fenA* and *fenB* genes. Each of the peptide synthetase contains an adenylation domain which recognizes and specifically adenylates aminoacids to form thioester bond with the cofactor 4′phosphopantetheine at the thiolation domain. The transpeptidation process extends the peptide length by transferring the activated amino acids in the initiation module to the activated amino acid in the thiolation domain in the next module. This process continues in a linear manner from one module to another till the entire molecule is constructed (Steller et al. 1999; Lin et al. 1999; Shu et al. 2002; Wu et al. 2007).

*Bacillus* sp. have already been reported to produce mixtures of closely related cyclic lipopeptide isoforms of the biosurfactant surfactin A (Snook et al. 2009). Among the many anticipated uses of surfactin, it is considered to be inhibitory to fungi, bacteria, mycoplasmas, and viruses (Bacon et al. 2012). The most remarkable feature highlighted with the surfactin is its broad range antibacterial activity. So the antibacterial basis of BmB 4 shown in the study can either be due to the activity of surfactin alone or its synergistic effect with fengycin. The lipopeptides extracted from *B*. *subtilis* UMAF6639 have been described to have iturin A, fengycin and surfactin as its chemical basis to inhibit spore germination (Marrone 2002). As lipopeptides are known to disrupt the microbial cytoplasmic membrane by creating transmembrane channels leading to the release of vital ions such as K^+^ (Hsieh et al. 2008), the result obtained in the study is very significant.

## Conclusion

From this study, endophytic *B. mojavensis* obtained from *B. monnieri* was confirmed for its potential to produce different classes of lipopeptides with broad spectrum antibacterial activity. As the antimicrobial potential of endophytic *Bacillus* species from medicinal plants are least investigated, the result of this study is significantly important and can have promising applications as the potential of lipopeptides is just began to explore.
